# Predictive performance of different NTCP techniques for radiation-induced esophagitis in NSCLC patients receiving proton radiotherapy

**DOI:** 10.1038/s41598-022-12898-8

**Published:** 2022-06-02

**Authors:** Mei Chen, Zeming Wang, Shengpeng Jiang, Jian Sun, Li Wang, Narayan Sahoo, G. Brandon Gunn, Steven J. Frank, Cheng Xu, Jiayi Chen, Quynh-Nhu Nguyen, Joe Y. Chang, Zhongxing Liao, X. Ronald Zhu, Xiaodong Zhang

**Affiliations:** 1 Department of Radiation Oncology, Ruijin Hospital, Shanghai Jiao Tong University School of Medicine, Shanghai, 200025 China; 2 Department of Radiation Physics, Unit 1150, The University of Texas MD Anderson Cancer Center, 1515 Holcombe Boulevard, Houston, TX 77030 USA; 3 Department of Radiation Oncology, Tianjin Medical University Cancer Institute and Hospital, Tianjin, 30060 China; 4 Department of Radiation Oncology, The University of Texas MD Anderson Cancer Center, Houston, TX 77030 USA; 5 Department of Experimental Radiation Oncology, The University of Texas MD Anderson Cancer Center, Houston, TX 77030 USA

**Keywords:** Radiotherapy, Non-small-cell lung cancer, Biomedical engineering

## Abstract

This study aimed to compare the predictive performance of different modeling methods in developing normal tissue complication probability (NTCP) models for predicting radiation-induced esophagitis (RE) in non–small cell lung cancer (NSCLC) patients receiving proton radiotherapy. The dataset was composed of 328 NSCLC patients receiving passive-scattering proton therapy and 41.6% of the patients experienced ≥ grade 2 RE. Five modeling methods were used to build NTCP models: standard Lyman–Kutcher–Burman (sLKB), generalized LKB (gLKB), multivariable logistic regression using two variable selection procedures-stepwise forward selection (Stepwise-MLR), and least absolute shrinkage and selection operator (LASSO-MLR), and support vector machines (SVM). Predictive performance was internally validated by a bootstrap approach for each modeling method. The overall performance, discriminative ability, and calibration were assessed using the Negelkerke R^2^, area under the receiver operator curve (AUC), and Hosmer–Lemeshow test, respectively. The LASSO-MLR model showed the best discriminative ability with an AUC value of 0.799 (95% confidence interval (CI): 0.763–0.854), and the best overall performance with a Negelkerke R^2^ value of 0.332 (95% CI: 0.266–0.486). Both of the optimism-corrected Negelkerke R^2^ values of the SVM and sLKB models were 0.301. The optimism-corrected AUC of the gLKB model (0.796) was higher than that of the SVM model (0.784). The sLKB model had the smallest optimism in the model variation and discriminative ability. In the context of classification and probability estimation for predicting the NTCP for radiation-induced esophagitis, the MLR model developed with LASSO provided the best predictive results. The simplest LKB modeling had similar or even better predictive performance than the most complex SVM modeling, and it was least likely to overfit the training data. The advanced machine learning approach might have limited applicability in clinical settings with a relatively small amount of data.

## Introduction

Radiotherapy is an essential treatment modality for patients with non-small cell lung cancer (NSCLC). However, radiation-induced toxicity, such as esophagitis, is of great concern to physicians as it lowers patients’ quality of life and may limit the dose-escalation targeted for better tumor control^1^. Accurate prediction of the normal tissue complication probability (NTCP) offers clinical guidelines for early intervention and facilitates physicians to develop a patient-specific treatment strategy. Moreover, there has been a growing interest in NTCP model-based patient selection for proton therapy^2–6^. For a given endpoint, modeling of NTCP can be performed using different approaches, ranging from the conventional analytic functions to the latest machine learning techniques^7^. And yet, there is no consensus on what is the best approach for NTCP modeling in the context of radiotherapy.

Traditional NTCP models were developed using analytic functions based on different assumptions of the dose–response relationship^8–13^. Among the analytic NTCP models, the Lyman–Kutcher–Burman (LKB) is the most well-known and widely used method^11–13^. In addition, it is the only NTCP model available in clinical treatment planning systems for biological evaluation^14^. In the standard LKB (sLKB) model, only the dosimetric factor was considered and the three-dimensional dose distribution was simplified into a single measure. It has been found the performance of the sLKB model was improved by adding one or more dose-modifying variables that account for the effect of non-dosimetric factors^15–17^. The LKB model with dose-modifying variables was denoted as the generalized LKB (gLKB) model hereafter. Another classic modeling technique to deal with various types of predictors is multivariable logistic regression (MLR), a statistical method to predict a binary outcome based on a set of independent variables^18^. Compared with the sLKB model which used only one dosimetric measure, the MLR model is capable of handling multiple dose-volume metrics, such as maximum dose, mean dose, V*x* values (the percentage volume receiving a dose greater than *x* Gy), and D*x* values (the minimum dose given to *x*% of the volume)^19^. The sLKB model showed a slightly lower discriminative ability than the MLR model^20^, but the difference between the performance of the generalized gLKB model and that of the MLR model has not been reported.

In the era of big data, the application of supervised machine learning in the treatment response modeling for radiotherapy has been rapidly growing. Support vector machine (SVM)^21^, a commonly used supervised machine learning algorithm, has shown promising accuracy in predicting lung radiation-induced pneumonitis^22^, local tumor control after stereotactic body radiation therapy for early-stage NSCLC patients^23^, and other clinical outcomes^24,25^. With the preset feature selection, the results of the comparison in performance between SVM and MLR were inconsistent^26,27^. It is of interest to compare their predictive power using a technique-specific feature selection scheme. Moreover, the advantage or the disadvantage of the complex SVM model over the simple LKB model remains unknown.

In this study, we aimed to identify the value of increasing the complexity of the NTCP model and using the advanced modeling technique in clinical practice by comparing the performance of LKB, MLR, and SVM methods for a data set concerning esophagitis.

## Materials and methods

### Data set

The data set consists of 328 NSCLC patients treated with passive-scattering proton therapy at The University of Texas MD Anderson Cancer Center during April 2006 to February 2012, and 136 (41.5%) patients experienced grade 2 or higher radiation-induced esophagitis^20^. This retrospective data collection was approved by our institutional review board (The University of Texas MD Anderson Cancer Center) with waivers for the patient’s informed consent. The prescription was 50–82.5 Gy [relative biological effectiveness (RBE)] in 25–37 fractions, with or without concurrent chemotherapy (CCRT). The clinical data were collected from the Epic system (Epic Systems Corporation), and the dosimetric data were extracted from the Eclipse treatment planning system (version 8.9, Varian Medical Systems) and has been converted to equivalent dose in 2-Gy fractions. Details of the patient characteristics, treatment, follow-up schedule, and esophagitis scoring were presented elsewhere^20^. The clinical characteristics included in the study were age (odds ratio (OR) = 0.99), sex (OR = 0.99), stage (OR = 2.23), and CCRT (OR = 4.76). There was no missing data on the outcome and predictors. All methods were performed in accordance with the relevant guidelines and regulations.

### Modeling techniques

#### Standard Lyman–Kutcher–Burman modeling

In the LKB model, NTCP is calculated using the probit formula coupled with a generalized equivalent uniform dose (EUD) dose-volume histogram-reduction scheme^28^. The sLKB model described the dose–response relationship characterized by three parameters, in which *n* represents the volume effect, *m* denotes the slope of the NTCP curve at *TD*_50_, and *TD*_*50*_ is the dose tolerance corresponding to 50% complication risk (Supplementary material 1).

#### Generalized Lyman–Kutcher–Burman modeling

In the gLKB model, a dose-modifying factor was introduced to account for the effect of clinical features. The use of CCRT has been shown to be associated with a higher probability of developing grade ≥ 2 esophagitis in this cohort of patients^20^. Therefore, we substituted the parameter *TD*_*50*_ with a group-specific *TD*_*50s*_ for the subgroup treated with (*TD*_*50y*_) or without (*TD*_*50n*_) CCRT, while keeping the same *n*, *m* for the entire cohort (Supplementary material 1). The dose-modifying factor is the ratio of *TD*_*50y*_ and *TD*_*50n*_.

In the sLKB and gLKB modeling, the same methods were used to determine the best fits and the 95% confidence intervals (CI) for the parameters. The maximum likelihood estimation was used to determine the optimal values for the parameters *n*, *m*, *TD*_*50,*_* TD*_*50y,*_ and *TD*_*50n*_. The 95% CIs for the fitted parameters were obtained using the profile likelihood method^29,30^.

#### Multivariable logistic regression

In the multivariable logistic regression model, the NTCP is modeled as a logit transformation of a linear function of several prognostic variables (Supplementary material 1). The candidate prognostic variables for each patient include four clinical and 17 dosimetric parameters. The clinical variables were sex, race, age, stage, and the use of CCRT. The dosimetric variables were EUD, the maximum dose (D_max_), the mean dose (D_mean_), and the percentage volume receiving a dose higher than *x* Gy(RBE) (*x* ranges from 10 to 75 in increments of 5). The results of the correlation between variables are available elsewhere^20^. Table 1 listed the encoding values of the clinical variables and the dosimetric values. The EUD was calculated using the parameter *n* derived for the sLKB model.

**Table 1:** Candidate clinical variables and EUD values of the data set.

Index	Variables	Range/classification	Mean/frequency
1	Sex	0, 1^I^	185, 143
2	Age, years	33–95	68.6
3	Stage	1, 2, 3, 4	59, 47, 208, 14
4	CCRT	0, 1^I^	130, 198
5	EUD, Gy(RBE)	0–68.02	37.63
6	D_max_, Gy(RBE)	0–86.00	62.91
7	D_mean_, Gy(RBE)	0–56.70	18.53
8	V10, %	0–93.54	37.44
9	V15, %	0–91.70	34.93
10	V20, %	0–90.23	32.48
11	V25, %	0–88.98	30.35
12	V30, %	0–87.89	28.28
13	V35, %	0–86.93	26.14
14	V40, %	0–86.06	23.93
15	V45, %	0–85.03	21.95
16	V50, %	0–83.15	19.82
17	V55, %	0–80.42	16.78
18	V60, %	0–73.00	14.01
19	V65, %	0–61.99	10.32
20	V70, %	0–59.12	7.12
21	V75, %	0–47.33	3.08

^I^0 = male, 1 = female.

^II^0 = no, 1 = yes.

*CCRT* concurrent chemotherapy, *EUD* equivalent uniform dose, *RBE* relative biological effectiveness, *D*_*max*_ maximum dose, *D*_*mean*_ mean dose, *V*_*x*_ percentage volume receiving dose higher than *x* Gy (RBE).

For the stepwise feature selection, a forward stepwise algorithm was adopted with the likelihood ratio test. Starting with a null model, the stepwise regression added or removed one variable at each step based on the *p* value for the likelihood ratio test. The variable selection process stopped if no variable can be added or removed. Stepwise-MLR is referred to as the multivariable logistic regression model using the stepwise feature selection in the following sections.

The LASSO method profiles out the insignificant variables by penalizing the regression coefficients of the variables and shrinks some of them to zero. As such, variables with non-zero coefficients were selected. The degree of penalty is controlled by the regularization parameter λ (Supplementary material 1). A fivefold cross-validation was performed to determine the optimal value of λ. The multivariable logistic regression model using LASSO regulation for feature selection is denoted as LASSO-MLR.

#### Support vector machine

For binary classification, the SVM searches for a hyperplane that maximizes the margin between the two classes. In this study, a radial kernel function was used to map the data from a low-dimensional space into a high-dimensional feature space where the non-linear boundary became a linear boundary. Two hyperparameters were included in the SVM model. C is the regularization parameter that trades off the margin width against the fitting error, and γ is the parameter in the kernel function that controls the overfitting. The NTCP estimates were then generated from a decision function using a logit transformation (Supplementary material 1).

A grid-research was performed in the space of (log_2_C =  − 5:2:15, log_2_γ =  − 3: − 2: − 15) to identify the optimal parameter pair (C, γ) using fivefold cross validation^31^. The whole data set was divided into 5 subsets of approximately equal size. In each iteration, a model was trained in 80% of the sample using a pair of (C,γ) and tested in the remaining 20% subsets. The procedure was repeated 5 times. The selection criterion was the averaged value of the area under the receiving operator curve (AUC) computed in the testing samples. All grid points of (C, γ) were tested and the one yielding the maximum AUC was picked. The best parameters of C and γ were then used in the feature selection. The optimal subset of features was determined by sequentially adding features based on the criterion of the fivefold cross-validated AUC until there is no improvement. In the SVM modeling, a total of 24 variables were included, where stage was partitioned as a four-category attribute (1,0,0,0), (0,1,0,0), (0,0,1,0), and (0,0,0,1).

### Bootstrapping for feature selection and model validation

The feature selection procedures in Stepwise-MLR, LASSO-MLR, and SVM were repeated in 1000 bootstrap samples drawn from the replacement of the original sample. The features with a selection frequency of greater than 80% were picked for the final models. Another threshold was applied for the selection frequency if the highest selection frequency was lower than 80%. With the selected features, models were built on the original sample and the performance was evaluated (apparent performance). For SVM, the grid-search step was repeated to find the best pair (C, γ) within the selected feature space.

We also used the bootstrap approach to internally validate the predictive performance of different NTCP models. Model fitting including feature selection and coefficient estimation was repeated on each bootstrap sample using the specific modeling technique. From the resulting model, the performance was evaluated in the bootstrap sample (bootstrap performance) and the original sample (test performance). The optimism was calculated as the difference between bootstrap performance and test performance. A lower value of optimism denotes a lower level of overfitting. The optimism should be corrected to reflect the stable and unbiased estimation of the model performance in future data. The optimism-corrected performance was then obtained by subtracting the optimism from the apparent performance.

The performance of the model was assessed using the Negelkerke R^2^, AUC, and Hosmer–Lemeshow (HL) test. Negelkerke R^2^ is an overall measure to quantify the variability explained by a model. AUC is used to indicate how well the model classifies patients into different risk groups. HL test assessed the calibration of the model, and a *p* value of greater than 0.05 indicates good agreement between predicted probability and observed risk.

### Software

Model development and data analysis were performed in MATLAB (version R2016b, MathWorks, Inc.). For the SVM modeling, we used the MATLAB version of the freely distributed package LIBSVM^32^.

## Results

In the gLKB model, the optimal values of *n* and *m* [*n* = 0.23 (95% CI: 0.09–0.47), *m* = 0.54 (95% CI: 0.41–0.74)] are slightly deviated from the values of sLKB model [*n* = 0.24 (95% CI: 0.10–0.49), *m* = 0.51 (95% CI: 0.37–0.70)]. The *TD*_*50*_ for patients treated with CCRT (*TD*_*50y*_) was 42.17 Gy (RBE) [95% CI: 31.76–52.53 Gy (RBE)], while it was 57.84 Gy (RBE) [95% CI: 42.65–79.75 Gy (RBE)] for patients treated without CCRT. Therefore, the dose-modifying factor is 1.4. Compared with the sLKB model, the gLKB model showed a better fit of the data, as can be deduced from the the increased value of optimism corrected Negelkerke R^2^ and AUC.

**Figure 1:**
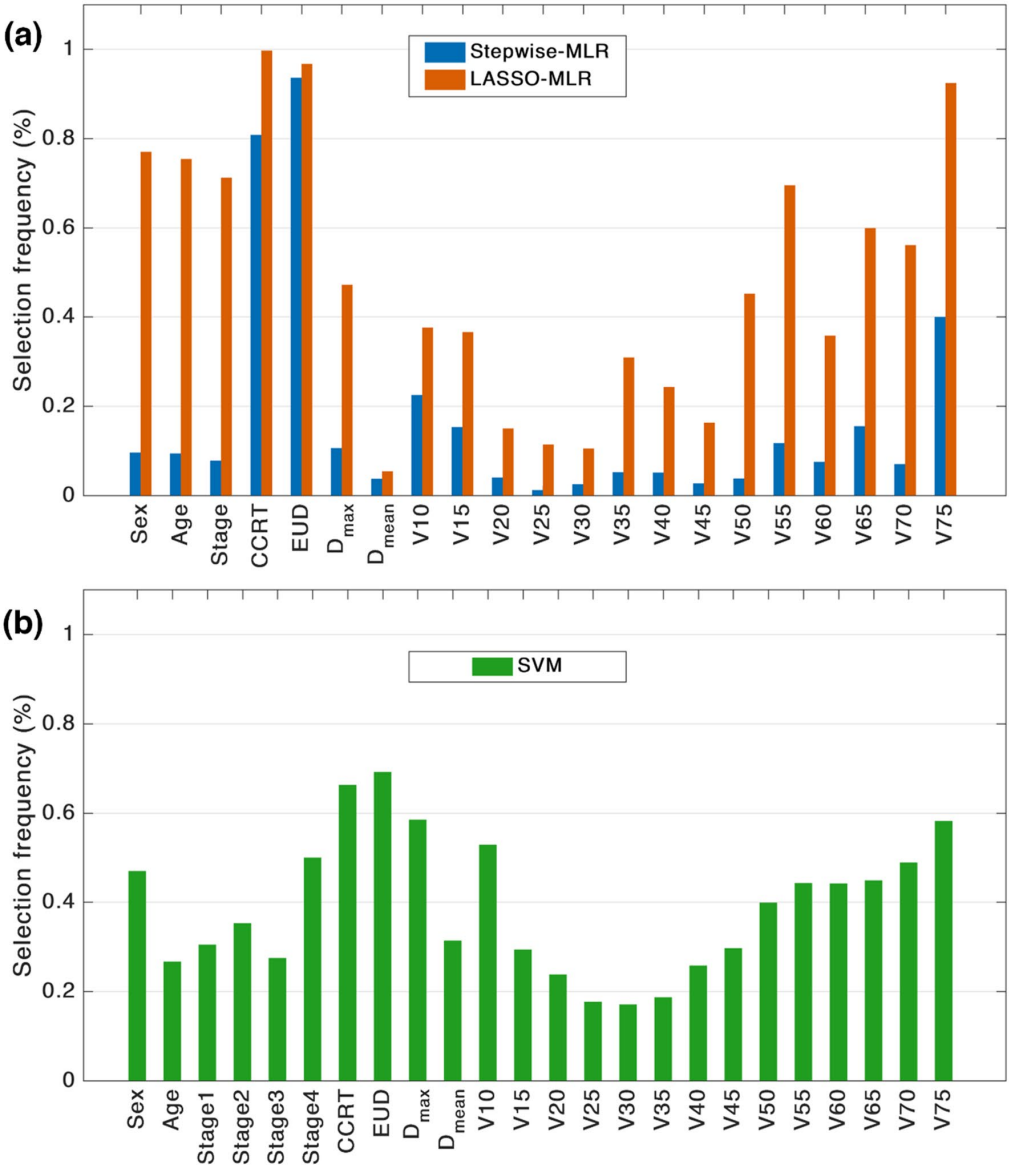
Selection frequencies of the candidate variables in 1000 bootstrapping samples by forward stepwise selection and LASSO for the MLR model (**a**), and by forward stepwise selection for the SVM model (**b**).

Figure 1 shows the selection frequency for all the variables in the 1000 bootstrap samples with the modeling techniques Stepwise-MLR, LASSO-MLR, and SVM. For the variables with greater than 80% frequency, stepwise selection and LASSO had two common variables: CCRT (stepwise: 80.8%, LASSO: 99.7%) and EUD (stepwise: 93.6%, LASSO: 96.7%). Compared with stepwise selection, LASSO tended to include more variables and it selected V75 with a frequency of 92.4%. Using 60% as a threshold, the variables selected by SVM were CCRT (66.3%) and EUD (69.2%), matching the variable selection based on the stepwise approach. Table 2 presents the regression coefficients for the two MLR models using the selected variables and the optimal values of C and γ for the SVM model.

**Table 2:** Feature selection results and parameter values for sLKB, gLKB, Stepwise-MLR, LASSO-MLR, and SVM models.

Models	Parameters/features	Coefficients/formula
sLKB	n, m, TD_50_	n = 0.24, m = 0.51, TD_50_ = 44.83 Gy (RBE)
gLKB	n, m, TD_50y_, TD_50n_	n = 0.23, m = 0.54, TD_50y_ = 42.17 Gy (RBE) TD_50n_ = 57.84 Gy (RBE)
Stepwise-MLR	CCRT, EUD	${\text{log}}\left(\frac{\text{p}}{{1}-{\text{p}}}\right)= -3.5845+0.8505*{\text{CCRT}}+0.0664*{\text{EUD}}$
LASSO-MLR	CCRT, EUD, V75	${\text{log}}\left(\frac{\text{p}}{{1}-{\text{p}}}\right)= -3.2766+0.7913*{\text{CCRT}}+0.0573*{\text{EUD}}+0.0438*{\text{V}}75$
SVM	CCRT, EUD	C = 2^15^, δ = 2^–13^

*sLKB* standard Lyman–Kutcher–Burman, *gLKB* generalized Lyman–Kutcher–Burman, *Stepwise-MLR* multivariable logistic regression using stepwise feature selection, *LASSO-MLR* multivariable logistic regression using least absolute shrinkage and selection operator for feature selection, *SVM* support vector machine, *CCRT* concomitant chemotherapy, *EUD* equivalent uniform dose, *V75* percentage of volume receiving dose higher than 75 Gy(RBE).

A summary of the apparent performance, bootstrap performance, optimism, and the optimism-corrected performance of all the models are listed in Table 3. The optimism-corrected Negelkerke R^2^ and AUC values of the LASSO-MLR were also higher than the other models. Stepwise-MLR showed a marginal improvement in the optimism-corrected-AUC (0.797) compared with the gLKB model (0.796). A similar predictive performance was found for the SVM and sLKB model with regard to the optimism-corrected Negelkerke R^2^ and AUC values. Both of the optimism-corrected Negelkerke R^2^ values of the SVM and sLKB models were 0.301, and the optimism-corrected AUC values were 0.783 and 0.784 for the sLKB and SVM models respectively. Moreover, there were minimal differences between SVM and gLKB, Of note, the advanced SVM modeling technique was more prone to overfitting, as indicated by its highest optimism (0.039 and 0.015 for Negelkerke R^2^ and AUC respectively). By contrast, the lowest optimism was observed in the conventional sLKB model.

**Table 3:** Apparent, bootstrap performance and optimism of sLKB, gLKB, Stepwise-MLR, LASSO-MLR, and SVM models.

Performance	sLKB	gLKB	Stepwise-MLR	LASSO-MLR	SVM
**Apparent**					
Negelkerke R^2^	0.315 (0.301)*	0.342 (0.323)*	0.344 (0.329)*	0.354 (0.332)*	0.340 (0.301)*
AUC	0.785 (0.783)*	0.799 (0.796)*	0.800 (0.797)*	0.803 (0.799)*	0.799 (0.784)*
HL test	χ^2^ = 12.01 (*p* = 0.24)	χ^2^ = 5.08 (*p* = 0.83)	χ^2^ = 5.18 (*p* = 0.79)	χ^2^ = 3.84 (*p* = 0.92)	χ^2^ = 5.60 (*p* = 0.78)
LL	− 178.55	− 174.46	− 174.20	− 172.48	− 174.75
**Bootstrap mean (95% CI)**					
Negelkerke R^2^	0.318 (0.210–0.427)	0.349 (0.244–0.454)	0.349 (0.246–0.452)	0.363 (0.260–0.465)	0.334 (0.207–0.460)
AUC	0.787 (0.737–0.836)	0.802 (0.753–0.850)	0.802 (0.753–0.850)	0.805 (0.757–0.853)	0.807 (0.756–0.858)
**Optimism mean (95% CI)**					
Negelkerke R^2^	0.014 (− 0.096–0.124)	0.020 (− 0.091–0.130)	0.015 (− 0.093–0.123)	0.022 (− 0.088–0.132)	0.039 (− 0.091–0.168)
AUC	0.002 (− 0.048–0.052)	0.004 (− 0.045–0.052)	0.003 (− 0.046–0.051)	0.004 (− 0.044–0.051)	0.015 (− 0.037–0.068)

*Apparent performance (optimism-corrected).

*sLKB* standard Lyman–Kutcher–Burman, *gLKB* generalized Lyman–Kutcher–Burman, *Stepwise-MLR* multivariable logistic regression using stepwise feature selection, *LASSO-MLR* multivariable logistic regression using least absolute shrinkage and selection operator for feature selection, *SVM* support vector machine, *AUC* area under the receiver operator curve, *HL* Hosmer–Lemeshow, *LL* log likelihood, *AIC* Akaike information criterion.

**Figure 2:**
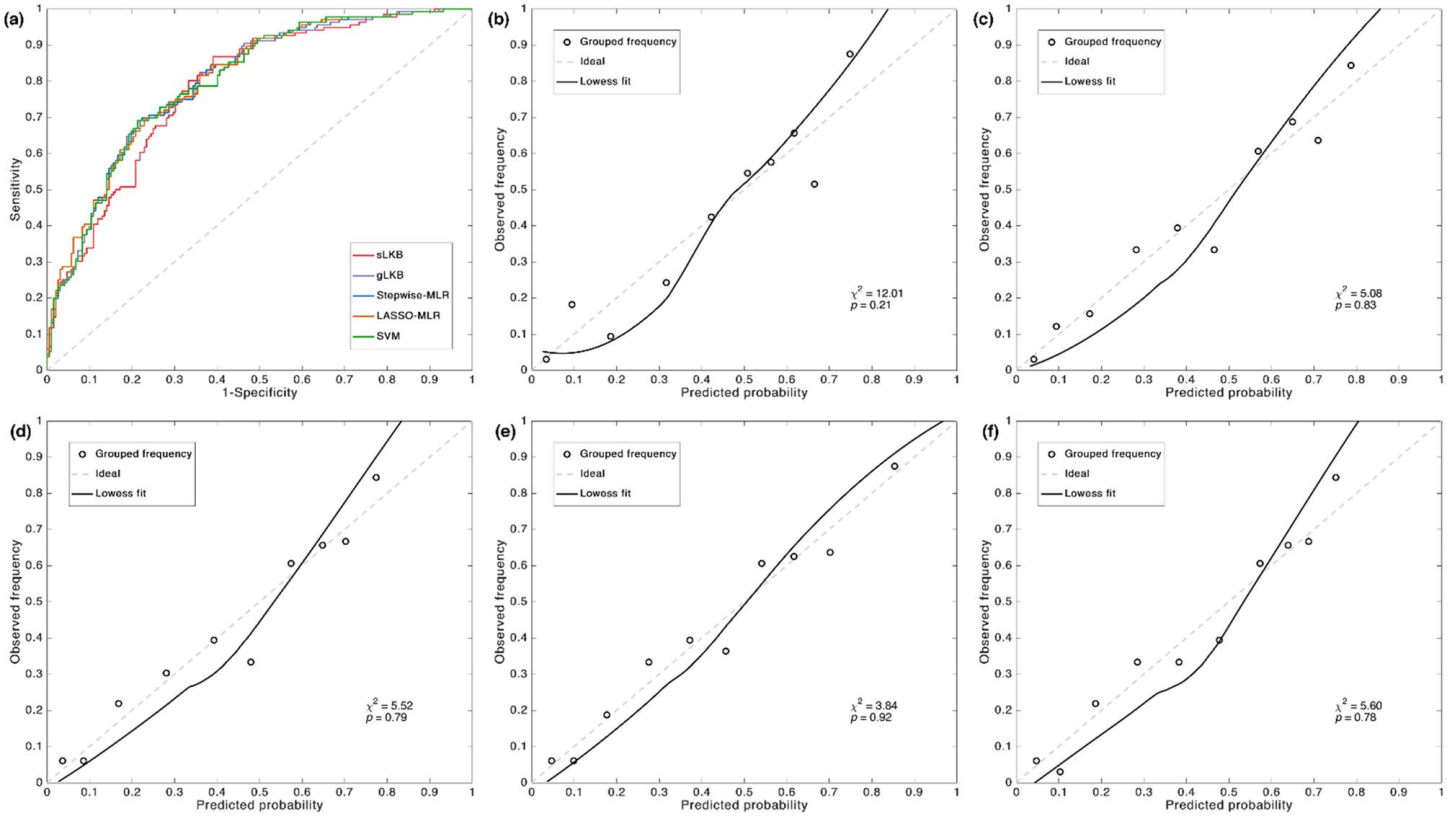
Receiver operator curves (**a**) and the calibration plot of actual outcome vs. predicted probability for the sLKB (**b**), gLKB (**c**), Stepwise-MLR (**d**), LASSO-MLR (**e**), and SVM (**f**) models. Results of the Hosmer–Lemeshow test are displayed in the lower right part of each plot. The open circles are the observed frequency in each group by the deciles of mean predicted probabilities. The dashed line represents the ideal prediction and the solid line is the loess fit for the output of the model.

Figure 2 displays the receiving operator curves and the calibration plots of all the models. A comparison of receiving operator curves of all the models (Fig. 2a) showed that sLKB was less accurate in detecting true positive cases at a higher threshold as compared with the other models. The predictions of all the models significantly agreed with the observed outcome, as demonstrated by the non-significant HL tests. The loess smoother of LASSO-MLR was closer to the ideal line, indicating a better calibration performance.

## Discussion

In this study, we investigated and internally validated the predictive performance of NTCP models developed for radiation-induced esophagitis in NSCLC patients receiving proton radiotherapy using different modeling techniques ranging from conventional analytic solution, logistic regression, to advanced machine learning. Of the five NTCP models, LASSO-MLR showed the best fit for the data. The sLKB model had the lowest optimism. SVM modeling resulted in NTCP models with a good apparent performance but with the highest optimism. Our results highlighted the non-inferiority of the conventional LKB modeling to the advanced SVM modeling in the discriminative ability and the accuracy of the predicted probability.

The predictive performance of the NTCP model was improved when more factors were considered. For the LKB modeling, our results of the comparison of the apparent performance were in line with the studies conducted by Peeter et al.^15^ and Defraene et al.^33^, who found the modified LKB model with both the dosimetry and clinical factors gave a significantly better fit than the standard LKB model with dosimetry alone for different endpoints. Previous studies also reported similar findings in multivariable logistic regression modeling that the model with more predictors had a higher performance than the one with fewer predictors^34,35^. However, the comparison of the optimism-corrected performance has not been investigated. Of note is that the optimism-corrected performance was considered a less unbiased assessment of the model in the future data than the apparent performance^36^. It is more likely to have a higher optimism by including more variables, thus resulting in lower optimism-corrected performance. Xu et al.^35^ demonstrated that an all-variable logistic regression model was susceptible to overfitting, which suggested a robust variable selection approach. As shown in Table 3, thought the optimisms of gLKB and LASSO-MLR were higher than those of their counterparts using the same modeling technique, the optimism-corrected performance of gLKB and LASSO-MLR were still higher.

The advanced SVM modeling technique did not offer improvement in our prediction of the dose–response relationship over the conventional LKB and logistic regression models. Moreover, the predictive performance of the SVM model was slightly poorer than the Stepwise-MLR that used the same predictors. The results would probably be explained by the γ parameter of the SVM model. In addition to the regulation parameter C, the behavior of the SVM model using the radial basis function kernel is very sensitive to the γ parameter. A large value of the γ parameter means that the radius of the influence area of the support vectors only includes the support vector itself and the regulation from the parameter C is unable to prevent overfitting. A small value of the γ parameter denotes an overly constrained model that cannot capture the complexity of the data, resulting in a model with a similar performance as the model assuming the linearity of the predictors. The γ parameter in the optimized SVM model has a relatively low value of 2^−13^. Therefore, the SVM model performed comparably to the logistic regression model under the same feature selection and even did not outperform the simplest analytic LKB model. Lynam et al.^26^ also reported that logistic regression performed as well as the machine learning algorithm in their study population when a small number of predictors were considered. Currently, there is no golden standard for variable selection and fewer significant variables are preferred in clinical practice. The nonlinear machine learning approaches may prove more powerful in the context of a larger dataset with more variables, such as the study of genomics and radiomics. In addition, the signal-to-noise of the clinical data may be lower. Therefore, the advanced machine learning modeling technique may have limited applicability in the current clinical setting.

For clinical practitioners, intuitive interpretability and ease of implementation were two important aspects when choosing the “best” model. The parameter *n* in the LKB model provides insight into whether the low dose region or the high dose region matters. We can also derive a dose constraint in terms of EUD from the LKB and logistic regression models based on the monotonically increasing functions of EUD, leading toward individualized treatment planning. In contrast, the mapping of input data and the output predictions in the SVM model is considered a “black box”, which prevents practitioners from better understanding the data and quantifying the effect of the dosimetric and non-dosimetric predictors. Incorporating the decision-making platform with a large amount of information given by the treatment planning system and the patient information system such as Epic would facilitate the adoption of a machine learning algorithm in clinical decision-making. In terms of performance and utility, LASSO-MLR was the best modeling technique in the current context.

One limitation of the current study is that only a few clinical predictors had been enrolled in the investigation. A further study would be different NTCP modeling techniques incorporated with functional imaging features and biological markers. In addition, larger and external data sets are needed to verify the findings of this study.

## Conclusion

In the context of classification and probability estimation for predicting the NTCP of radiation-induced esophagitis in NSCLC patients receiving proton radiotherapy, a multivariable logistic regression model developed with LASSO provided the best predictive results. The simplest LKB modeling using the analytic function had similar or even better predictive performance than the most complex SVM modeling, and it was least likely to overfit the training data. The advanced machine learning approach might have limited applicability in clinical settings with a relatively small amount of data.

## Supplementary Information

Supplementary Information.

## Data Availability

The datasets used and/or analysed during the current study available from the corresponding author on reasonable request.
