# Supplementary material for: Predictive performance of different NTCP techniques for radiation-induced esophagitis in NSCLC patients receiving proton radiotherapy
Source: Sci Rep. 2022 Jun 2;12:9178. doi: 10.1038/s41598-022-12898-8 (PMC9163134; doi:10.1038/s41598-022-12898-8)
Supplement: Supplementary file 1 — Supplementary Information. [file 41598_2022_12898_MOESM1_ESM.docx]

**Supplementary Material 1**

### Standard Lyman-Kutcher-Burman modeling

In the standard Lyman-Kutcher-Burman(sLKB) model, normal tissue complication probability (NTCP) is calculated as follows:

$\mathrm{NTCP}=\frac{1}{\sqrt{2\pi}}\int_{-\infty}^{t} e^{-\frac{x^{2}}{2}}dx$,

where the integral limit *t* is given by

$t=\frac{\mathrm{EUD}-{TD}_{50}}{m\times{TD}_{50}}$

and

$\mathrm{EUD}={(\sum_{i} v_{i}{D_{i}}^{\frac{1}{n}})}^{n}$,

where *v_i_* is the relative volume of each dose bin *D_i_*.

### Generalized Lyman-Kutcher-Burman modeling

The generalized LKB (gLKB) model applied the same integral formula as the sLKB model, but the integral limit *t* was modified as

$t=\frac{\mathrm{EUD}-{TD}_{50s}}{m\times{TD}_{50s}}, {TD}_{50s}={TD}_{50y} or {TD}_{50n}$ .

where *TD_50y_* and *TD_50n_* are the *TD_50_* for the group of patients with or without concurrent chemotherapy respectively.

### Multivariable logistic regression

In the multivariable logistic regression model, the NTCP is modeled as a logit transformation of a linear function of *s* prognostic variables (*x_i_*).

$$NTCP=\frac{1}{1+e^{-f(x_{i})}}$$

$$\text{f(}x_{i}) \text{= }b_{0}+\sum_{i=1}^{s} b_{i}x_{i}, i=1,\ldots,s$$

where the intercept $b_{0}$ and the variable coefficients $b_{i}$ were determined by the maximum likelihood estimation.

In the likelihood ratio test for the stepwise feature selection, the likelihood ratio (LR) was calculated as

$$LR=-2log\frac{L_{fit}}{L_{s}}$$

where

$${LL}_{fit}=\sum_{i=1}^{N} y_{i}\ln\left( {NTCP}_{i} \right)+\left( 1-y_{i} \right)\ln\left( 1-{NTCP}_{i} \right)$$

in which $L_{fit}$ , $L_{s}$ are the likelihoods of the fitted model and saturated model, respectively. N is the number of cases, and $y_{i}$ is the binary outcome (y = 0: no grade ≥2 esophagitis, y = 1: grade ≥2 esophagitis).

The LASSO regression search for the best fits of regression coefficients that minimizing

$$\frac{1}{N}Deviance\left( b_{0},b \right)+\lambda\sum_{i=1}^{s} \left| b_{i} \right|$$

in which N is the number of cases, *s* is the number of variables. The degree of penalty is controlled by the regularization parameter λ. All the components of *b* are non-zero when λ equals to zero, and the number of non-zero components of *b* decreases as λ increases.

### Support vector machine

Given *l* training vectors **x***_i_*, *i* = 1, …, *l*, and *y_i_* = ± 1, SVM is optimized to minimize the cost function

$$\frac{1}{2}\boldsymbol{\omega}^{T}\boldsymbol{\omega}+ C\sum_{i=1}^{l} \xi_{i}$$

subject to

$y_{i}\left( \boldsymbol{\omega}^{T}\phi\left( \boldsymbol{x}_{i} \right)+b \right)\geq1-\xi_{i}$,

$\xi_{i} \geq0,i=1,\ldots,l$,

where $\boldsymbol{\omega}$ is the normal vector to the hyperplane and $\phi\left( \boldsymbol{x}_{i} \right)$ is the mapping function. C >0 is the regularization parameter that trades off the margin width against the fitting error, and $\xi_{i}$ are slack variables. $K\left( \boldsymbol{x}_{i},\boldsymbol{x}_{j} \right)= {\phi\left( \boldsymbol{x}_{i} \right)}^{T}\phi\left( \boldsymbol{x}_{j} \right)$ is the kernel function. The radial kernel function was calculated as:

$$K\left( \boldsymbol{x}_{i},\boldsymbol{x}_{j} \right)=e^{(-\gamma{\left\| \boldsymbol{x}_{i}-\boldsymbol{x}_{j} \right\|)}^{2}}$$

where $\gamma>0$ is a user-specified parameter. The NTCP estimates were then generated from a decision function using a logit transformation.

$$NTCP=\frac{1}{1+e^{(Af\left( \boldsymbol{x} \right)+B)}}$$

the decision function is given by

$$f\left( \mathbf{x} \right)=\sum_{i=1}^{l} \alpha_{i}y_{i}K(\boldsymbol{x},\boldsymbol{x}_{\boldsymbol{i}})+b$$

where $\alpha_{i} \geq0$ are the Lagrange multipliers. The parameter A and B are estimated by maximizing the log likelihood of the training data.
